# Perceived Usefulness of Smartphone Medical Apps As Theoretical and Clinical Learning Aids Among Medical Students in Pakistan: A Cross-Sectional Study

**DOI:** 10.7759/cureus.41682

**Published:** 2023-07-11

**Authors:** Hira Khan, Syed Waqas, Shilpa Golani, Muhammed M Kadir, Mohammad Ashraf

**Affiliations:** 1 Department of Community Health Sciences, Aga Khan University Hospital, Karachi, PAK; 2 Department of Neurological Surgery, Wolfson School of Medicine, University of Glasgow, Glasgow, GBR

**Keywords:** learning in low- and middle-income countries, clinical year medical students, digital learning, smartphone applications, medical education

## Abstract

Background

Smartphone applications have become popular tools in clinical educational environments, particularly because they enhance learning in any setting through their accessibility. Despite students utilizing these apps in their daily learning, Pakistan’s medical education system has yet to strongly endorse them. Given the rising usage of medical applications among clinical year medical students and the wide range of apps accessible on contemporary devices aimed specifically at the student population, there is a lack of literature addressing the use of these apps on clinical learning in low- and middle-income countries (LMIC) such as Pakistan.

Objectives

Our study aims to (1) assess the level of awareness among clinical-year medical students in Pakistan, of smartphone applications for academic purposes, (2) determine the usefulness of medical apps as educational tools for clinical-year medical students, in terms of enhancing overall patient-care skills and (3) identify barriers to the usage of apps among students who do not have them installed.

Methods

This online questionnaire-based study includes clinical year medical students across four medical colleges (two private and two public sectors) in Pakistan. Participant identity was kept anonymous and informed consent was required to participate. A sample size of 360 was used based on previous studies in the UK and student estimates from chosen medical colleges. The questionnaire tool used consists of three sections; demographics and medical school information, perceived usefulness of medical smartphone apps on a Likert Scale and barriers to usage among students who do not have them installed.

Results

97.9% of the total study population chose to participate in the study. There was roughly an equal percentage of responses from each clinical year and 72% of students reported active use of medical apps of which the vast majority (48%) have one to two apps on their phones. Only 39% of students felt that their medical colleges encourage the use of smartphone apps for academic purposes. 54% of students use apps to look up medical criteria for disease processes and almost 42% use them to search medications. On a Likert scale of 1-5, improvement of clinical performance received highest average score among users (3.92, SD 1.1), followed by quick access to medical guidelines (3.83, SD 1.0). The most common reasons for nonuse of medical apps were medical colleges not offering subscriptions and not knowing how to utilize apps.

Conclusion

Smartphone apps are widely used by clinical year medical students for academic purposes in our study. Despite lack of endorsement from their respective medical colleges, these apps are still popularly utilized for revision and research on disease criteria during clinics and rounds. Encouragement from the university has been identified as a significant barrier, however. Students who use smartphone apps reported an improvement in clinical performance overall; they were able to retrieve information quicker during rounds and noticed enhancements in formulating diagnoses and reading radiological images. In contrast, those not using these apps faced challenges with interpreting imaging results, recalling pharmacological properties of medications and developing differential diagnoses. Through these findings, we highlight the benefits of incorporating technological media into the undergraduate curriculum and hope medical universities from Pakistan can take inspiration.

## Introduction

The use of mobile applications has become a crucial aspect in the field of medical education, as healthcare professionals and students alike utilize these innovative technologies as a part of their training and practical experience. These applications have become indispensable in clinical educational environments, particularly due to their accessibility and ability to facilitate learning from any location [1]. Many experts believe that the integration of mobile learning initiatives will greatly improve the educational processes in healthcare. Several studies have been conducted to evaluate the impact of mobile learning on knowledge, skills, attitude, and satisfaction, but these findings have not been rigorously scrutinized, and there is currently a lack of quantifiable evidence on the effectiveness of mobile learning interventions [2]. Previous research indicates a significant majority of healthcare professionals and medical students, over 85%, utilize smartphones, and a substantial proportion, between 30% and 50%, utilize medical applications for purposes of learning and obtaining information [3].

Medical students have indicated that they utilize mobile applications in all stages of patient care learning, encompassing the collection of patient history, physical examination, diagnostic tests, medication prescription, and clinical management [4]. Despite being widely viewed favorably by medical professionals, mobile applications have yet to be fully integrated as a fundamental component of medical education, especially in resource-limited regions such as Pakistan [5]. Medical institutions globally have fostered the utilization of medical apps by taking advantage of group subscription options offered by various apps, making them accessible to their students. With the increasing utilization of these apps by medical students, there is a growing need for an evaluation of their effectiveness in enhancing the students' patient approach and differential diagnosis skills.

Given the rising usage of medical applications among clinical year medical students and the wide range of apps accessible on contemporary devices aimed specifically at the student population, there is a lack of literature addressing the effect of these apps on clinical learning in low- and middle-income countries (LMIC) such as private and public medical colleges in Pakistan. The literature review reveals that there is a scarcity of research conducted to assess the effectiveness of smartphone medical apps as learning aids for clinical year students in Pakistan. Therefore, our study aims to (a) assess the level of awareness among clinical year medical students in Pakistan, of smartphone applications for academic purposes, (b) determine the usefulness of medical apps as educational tools for clinical year medical students, in terms of enhancing overall patient-care skills and (c) identify barriers to usage of apps among students who do not have them installed for academic purposes.

## Materials and methods

We conducted an online questionnaire-based cross-sectional study that included four medical schools in Pakistan with the above-mentioned objectives and aims. The study received a full Ethical Review Committee (ERC) exemption at the Aga Khan University (AKU) Hospital, which permitted the survey to be conducted during work hours. Participation in the study was voluntary and anonymous, and full informed consent was taken prior to participation. No incentives or payments were given for taking part in the study. Data was collected using convenience sampling, and the survey was distributed from January 1, 2023 to March 1, 2023 through a confidential online questionnaire platform (Google Forms). The sample was selected from two public and two private medical colleges in Pakistan, namely AKU, Dow University of Health Sciences (DUHS), CMH Lahore Medical College and Institute of Dentistry, and King Edward Medical University (KEMU).

Participants 

In this cross-sectional study, the participants were medical students (MBBS) in clinical years three, four, and five from the selected medical schools in Pakistan and above the age of 18. Medical students who were below the age of 18 or who did not provide informed consent were excluded from the study. The sample size, calculated using OpenEpi, was determined to be at least 360 clinical-year medical students, with a 95% confidence interval and a precision of 5%. The sample size estimate was based on clinical year medical student estimates from chosen medical colleges, taking into account batch size and gender ratios. A total of 360 participants were approached, out of which eight declined to participate. Ultimately, data were collected from 352 participants, of which 203 (57.7%) were females and 147 (41.8%) were males. The average age of the participants was 22.3 years.

Questionnaire tool

A Google Forms-based questionnaire was designed using pre-existing literature modified to suit the needs of our study. The questionnaire tool used consisted of three sections: section 1; demographics, medical school information, and frequency of use of apps, section 2; perceived usefulness of medical smartphone apps in different clinical performance parameters on a Likert Scale of 1-5 and Section 3; barriers to usage among students who do not have them installed and clinical performance parameters these students struggle with the most.

Apps were divided into three categories broadly based on their main function. Medical reference and knowledge apps [3,6]; which allow access to up-to-date guidelines, research, and in-depth topic discussions, allowing the user to sift through relevant and informative medical data regardless of time and place. Medical calculator apps [7]; contain specialized tools to calculate different patient medical scores supported by evidence-based medicine, diagnostic criteria, formulae, and dosing calculations. These apps also aid in performing risk stratification of patients in a clinical setting. Lastly, clinical diagnosis-making support apps [8]; assist in formulating a list of potential differential diagnoses based on clinical signs and patient presentation. Moreover, several of these apps provide supportive radiological evidence as references and expand upon possible treatment options and patient-approach strategies [9-11].

Data collection

The online survey was distributed by recruiting collaborators in each medical school who facilitated communication between the lead authors of the study and the clinical year medical students. The questionnaire was shared with the students via popular social media platforms, such as WhatsApp, LinkedIn, and email. The participation of the students was voluntary, and informed consent was obtained from all participants. The survey was conducted on an anonymous basis, and participants were informed that the data would be utilized solely for scientific purposes.

Analysis

Analysis was done on SPSS Version 27 and Microsoft Excel. The mean and standard deviation of the values were calculated.

## Results

About 97.8% of the total study population chose to participate in the study. There was roughly an equal percentage of responses from each clinical year (years 3-5) and 57% of responses were from public sector medical colleges whereas 43% were from private sector medical colleges.

Two hundred fifty-five students (72.4%) reported using smartphone medical apps of which 22% reported daily use, followed by two to three times weekly use by 20% of the students. Only 39% of students felt that their medical colleges encourage the use of smartphone apps for academic purposes as shown in Table 1.

**Table 1:** Demographic data of students from four medical colleges and the awareness of the use of medical apps It includes median age with interquartile range, gender, college names and sector, smartphone ownership, usage of medical apps, college endorsement of app subscriptions, and frequency of app usage.

Parameter	Frequency (N)	Percentage (%)
Age (Median/IQR)	22/1	-
Gender		
Male	147	41.8
Female	203	57.7
Name of medical college		
King Edward Medical College	139	39.5
Aga Khan University Hospital	62	17.6
Dow University of Health Sciences	62	18.2
CMH Lahore	87	24.7
Medical College Sector		
Public	202	57.4
Private	150	42.6
Years of Medical College		
3^rd^ Year	108	30.7
4^th^ Year	106	30.1
5^th^ year	138	39.2
Ownership of smartphone		
Yes	358	100
No	0	0
Use of medical apps		
Yes	255	72.4
No	97	27.6
Does your medical college endorse medical apps and provide subscriptions to you?		
Yes	138	39.2
no	214	60.8
Frequency of use of medical apps		
Daily	77	21.9
Once a week	47	13.4
2-3 times a week	70	19.9
Once a month	52	15.4
Less than once a month	106	30.1

The most popular categories of apps are medical knowledge and reference apps (81%) followed by medical score calculator apps (27.9%) as shown in Table 2. One hundred seventy students (48.3%) had one to two apps installed on their phones and the most popular uses of apps were to revise clinical topics (69%), look up medical criteria for disease processes (54%) followed by searching up medications (41.9%) as shown in Table 3. More than one option could be selected by the respondents.

**Table 2:** Frequency of use of medical app categories The medical apps are divided into three different categories and an “others” option. N: Frequency

Category of apps	N	%
Medical reference and knowledge apps (Medscape, UpToDate etc)	209	81
Medical calculator apps (MDCalc etc, Pharmapedia)	72	27.9
Clinical diagnosis-making support apps (VisualDx)	37	14.3
Others	23	6.74

**Table 3:** Number of medical apps being used by students and purpose of use N: Frequency

Parameter	N	Percentage (%)
Number of medical apps installed		
None	94	26.7
1-2	170	48.3
3-4	59	16.8
>4	29	8.2
Purpose of use		
Calculate medical scores	50	19.4
Look up medical criteria for a specific disease process	140	54.3
Revision of clinical topic	178	69
Look up radiological imagery	84	32.6
Accessing information during rounds	95	36.8
Look up physical examinations	79	30.6
Search medications	108	41.9
Search differentials based on clinical presentation	105	40.7
Revision of medical content	14	1.6
I don’t use them	4	1.55

On a Likert scale of 1-5, improvement of clinical performance received the highest average score among users (3.92, SD 1.1), followed by quick access to medical guidelines (3.83, SD 1.0) and a better understanding of the pathophysiology of disease processes (3.76, SD 1.06). Overall, all clinical performance parameters showed an average score of above 3.0 as shown in Table 4.

**Table 4:** Impact of medical app usage on clinical performance The table displays the different avenues of the usefulness of medical apps and how they enhance medical education for clinical year students. The responses were measured on a Likert Scale - 1 = strongly disagree, 2 = disagree, 3 = not sure, 4 = agree, and 5 = strongly agree. SD: Standard Deviation

Parameter	Mean of scores (1-5)	SD
Medical apps help me understand the pathophysiology of a disease process	3.76	1.06
Medical apps help me learn clinical skills and examinations better	3.57	1.08
Medical apps help me quickly access medical guidelines during clinic/rounds	3.83	1.05
Medical apps help me formulate differential diagnoses	3.52	1.07
Medical apps help me understand radiological imagery better	3.52	1.12
Medical apps help me revise medical topics	3.70	1.16
I have noticed my clinical performance improve since I started using medical apps	3.92	1.13
Medical apps have helped me refine my patient approach skills	3.57	1.02
I would recommend the use of medical apps to other medical students	3.30	1.09

The most common reasons for nonuse of medical apps were medical colleges not encouraging them/ not offering subscriptions (37.2%) and not knowing how to fully use the applications in their academic learning (59.6%). These factors are reflected in Table 5.

**Table 5:** Reasons for nonuse of medical apps The table shows the barriers clinical year medical students face in accessing and utilizing medical apps for their education. More than one option could be selected by the respondents. N: Frequency

Factors contributing to your non-use of medical apps	N	%
My medical college does not encourage them/ does not offer subscriptions	35	37.2
I do not know how to utilize them	56	59.6
I believe they will not make a significant impact on my learning	10	10.6
None of my colleagues use them	16	17

**Figure 1:**
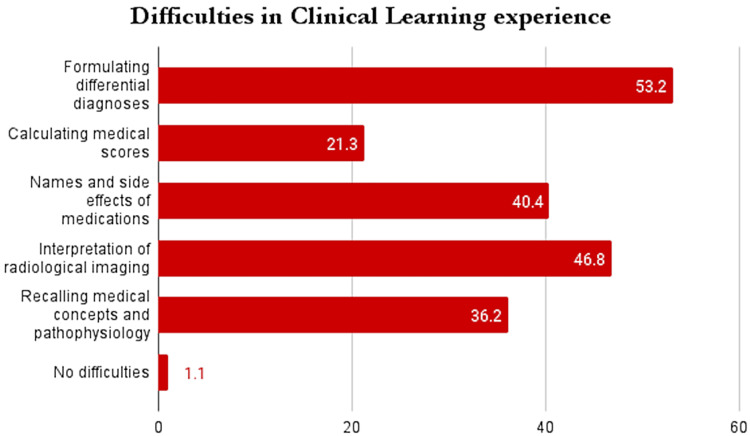
Difficulties in clinical learning experienced by students who do not utilize medical apps The values signify the percentages (%).

Among students who did not use apps, the majority reported facing the most difficulty in formulating differential diagnoses followed by interpreting radiological imaging, both of which are crucial clinical performance parameters in clinical years of medical school (Figure 1).

## Discussion

The present study in Pakistan, an LMIC, marks the first attempt to explore the perceived usefulness of medical smartphone apps among clinical year students in the country. With a focus on four leading medical universities across two different cities, this investigation encompasses both public and private institutions. By incorporating respondents from diverse socio-economic backgrounds, the research aims to enrich its data pool with heterogeneous perspectives.

The present investigation enlisted clinical year students from various universities. The response rate, estimated by dividing the number of participants by the total count of eligible candidates in each batch, was found to be around 17.8%. Notably, a previous study conducted in Canada [12] involving medical students also reported a low response rate (14%). This drawback may be attributed to “survey fatigue” and “non-response” bias that could have arisen due to the use of an online questionnaire format [13].

In our investigation, it was observed that every participant possessed a smartphone. This is unsurprising given Pakistan's high mobile penetration rate of roughly 90%, which surpasses all other countries in the South Asia region [14]. Comparable research in Pakistan [14] also discovered that each of its respondents owned a smartphone indicating the widespread use of smartphones among medical students and suggesting an opportunity to incorporate novel phone-based learning methods.

In terms of gender distribution among participants who responded to the questionnaire, 57.7% were female while male respondents were outnumbered. This could be due to women constituting around 70% of medical students nationwide in Pakistan as reported by studies previously conducted on Pakistani healthcare education demographics [15]. As such incorporating features specifically tailored towards females or implementing marketing strategies targeting them could prove valuable for future advancements made regarding applications centered around medicine on smartphones.

The focus of our investigation was restricted solely to students in their clinical years, as they exhibit a heightened level of enthusiasm for their medical education during this phase, attributable to the realistic situations presented and consequential higher stakes involved. Correspondingly, an antecedent study [14] corroborated that fourth- and fifth-year pupils were more prone to employing smartphones as tools for obtaining guidelines, medication details, and educational materials due to analogous rationales stated earlier.

The growing prevalence of smartphone and app adoption has impacted the medical field. Medical professionals and students have demonstrated a heightened interest in app usage, leading to the creation of a specialized medical app category on the Apple App Store in 2008 [16]. This trend is also reflected in our study as 72.4% of the responders use medical applications for their clinical rotations, with almost 21.9% using it every day.

From the results of our study, 81% of the students use medical reference and knowledge apps such as Medscape and UpToDate. This is not an isolated finding but similar to a previous study [13], which can be accredited to the better user interface, updated clinical information, and quality medical content of these applications. Moreover, university faculty have recommended these same resources as additional learning aids for medical students at large [17].

From our study, we found that almost 48.3% of respondents had one to two medical applications installed compared to only 8.2% who had greater than four apps. This limit on the number of apps can be related to multiple factors presenting as an obstacle to the usage of these apps. In LMIC where education and awareness regarding technology within the community are still scarce, many students may find using their phones in front of the patients and attendants as unprofessional [18]. This stigma may also be perpetuated by senior faculty who may deem using medical apps in clinical settings with a negative perception and would instead have students resort to traditional books.

A study conducted in the University of Ottawa, Canada, among medical students, and residents regarding mobile app use [19] highlighted a few barriers that can be applied in our setting too. These include the cost of medical app subscriptions, which is a genuine issue in LMIC as students are already suffering from the increasing inflation rates. Poor Wi-Fi coverage inside the hospital and lack of knowledge on which apps to use are important factors that must be taken into consideration too. Providing high-speed internet services would be the institution’s responsibility, which can be influenced by high-quality studies illuminating the efficacy of the medical apps in improving education as well as clinical outcomes. Guidance provided to medical students by seniors and faculty about the recent innovations and updates in medical apps, and how to use them as an accurate source of information could help mitigate the “lack of knowledge” factor.

We also evaluated the purpose of the use of medical apps among clinical year students, in which “revision of clinical topic” was the most sought-after option. A similar study [20] conducted among 448 medical students in Pakistan published in 2018 found 48% to also be of the same view. Through quick access to information provided by medical apps, relative to textbooks, students may find revision of certain topics more convenient during the waiting periods of their rotations. “Looking up Medical criteria” was also a popular option selected by the respondents, a finding which is comparable to another similar study conducted in the US which found that 78% of medical students agreed that medical apps improve diagnostic accuracy [21].

Through our investigations, we assessed the impact of medical apps clinical performance of the students. The mean average for all the questions recorded on a Likert scale amounted to greater than 3, indicating a positive response among the students. The questions included in this assessment were if the apps bettered their understanding of the disease, refined their clinical skills, and if the students will recommend these apps to others. This finding can be compared to another study that also assessed the impact of a medical app among medical students from the Brighton and Sussex Medical School [22]. They found a significant association between the usage of that app by students and better performance in exams.

The 94 participants who do not use medical apps completed a separate section of the questionnaire, assessing factors which have led to their disuse. Almost 60% felt not know how to utilize smartphone medical apps. This could stem from multiple reasons such as untrustworthiness toward the medical apps or finding comfort within traditional books as resources. Also, 37.2% of the respondents are unable to access these apps as their medical colleges do not offer subscriptions. This barrier is highlighted in our study, as students may find it to difficult pay hefty prices for these applications during their undergraduate studying, especially in a region such as Pakistan where it is not a cultural norm for students to hold paying part-time jobs.

The results of this study have helped establish an outline for the usage and demand of smartphone apps as clinical learning aids among medical students in the selected public and private medical colleges in Pakistan. Although endorsement of apps by medical colleges may be highlighted as a barrier for most medical students, they continue to use them due to the positive impact they have experienced in their undergraduate learning using a wide range of apps. This study also highlights the need for more research to be done in this area to improve clinical learning outcomes and can be taken a step further to see a numerical change in clinical rotation scores with the incorporation of these apps.

There were certain limitations to our study that we could identify. Firstly, our sample size was small including only four institutions out of the many that are present in Pakistan, thereby not adequately covering the entire demographics. Secondly, we used an online survey form which led to a reduced response rate, possibly due to volunteer bias. Our data collection method may also be susceptible to other types of bias as there is no face-to-face engagement between the survey administrator and respondent to elucidate queries regarding the questionnaire. Also, since the nature of the form was digital, it is more likely that the respondents are avid users of technology with positive re-enforcements towards innovating medical education. Moreover, our study did not investigate the interests of medical students towards each specialty and how that can influence the usage of medical applications. Our study can be used as a basis to investigate the topic further and explore the potential drawbacks of using technology in an education setting such as students encountering inaccurate or outdated information and potential over-reliance on technology as learning aids.

## Conclusions

Most clinical year medical students in the scope of our study continue to utilize smartphone apps for academic purposes, regardless of endorsement by their respective medical colleges; however, lack of endorsement has been identified as a significant barrier in our study to the use of apps. The most popular use of apps was for revision purposes and to look up clinical criteria for disease processes in order to make clinical decisions and differential diagnoses. Students reported noticing an improvement in overall clinical performance after using smartphone apps, could retrieve information quicker during clinics and rounds, and felt an improvement in formulating clinical diagnoses and reading radiological imagery. Students not utilizing smartphone apps for academic purposes reported the most difficulty in formulating differential diagnoses, interpreting radiological imaging, and recalling the pharmacological properties of medications. Our study shows the positive impact of smartphone apps on clinical learning and performance in clinical year medical students in Pakistan and suggests that stronger endorsement and integration of technology into the undergraduate curriculum from medical colleges may significantly help future physicians excel in their undergraduate education.
